# Performance of Rice Husk Ash as Supplementary Cementitious Material after Production in the Field and in the Lab

**DOI:** 10.3390/ma13194319

**Published:** 2020-09-28

**Authors:** Mareike Thiedeitz, Wolfram Schmidt, Michelle Härder, Thomas Kränkel

**Affiliations:** 1 Centre for Building Materials, Technical University of Munich, 81245 München, Germany; michelle.haerder@tum.de (M.H.); thomas.kraenkel@tum.de (T.K.); 2 Bundesanstalt für Materialforschung und -prüfung, 12205 Berlin, Germany; Wolfram.Schmidt@bam.de

**Keywords:** rice husk ash, agricultural by-products, supplementary cementitious materials, waste management, carbon dioxide emissions

## Abstract

Supplementary cementitious materials (SCM) can reduce the total amount of Portland cement clinker in concrete production. Rice husk ashes (RHA) can be converted from an agricultural by-product to a high-performance concrete constituent due to a high amount of reactive silica with pozzolanic properties if they are burnt under controlled conditions. The way and duration of combustion, the cooling process as well as the temperature have an effect on the silica form and thus, the chemical and physical performance of the RHA. Various studies on the best combustion technique have been published to investigate the ideal combustion techniques. Yet, the process mostly took place under laboratory conditions. Investigating the difference between the performance of RHA produced in a rural environment and laboratory conditions is useful for the assessment and future enhancement of RHA production, and its application both as building material, for example in rural areas where it is sourced in large quantities, and as additive for high performance concrete. Thus, the paper presents a comparison between RHA produced under rudimentary conditions in a self-made furnace in the rural Bagamoyo, Tanzania and under controlled laboratory conditions at the Technical University of Munich, Germany, with different combustion methods and temperatures. In a second step, RHA was ground to reach particle size distributions comparable to cement. In a third step, cement pastes were prepared with 10%, 20% and 40% of cement replacement, and compared to the performance of plain and fly ash blended cement pastes. The results show that controlled burning conditions around 650 °C lead to high reactivity of silica and, therefore, to good performance as SCM. However, also the RHA burnt under less controlled conditions in the field provided reasonably good properties, if the process took place with proper burning parameters and adequate grinding. The knowledge can be implemented in the field to improve the final RHA performance as SCM in concrete.

## 1. Introduction

Concrete production and construction contribute to global carbon emissions and therefore climate change in many ways. Without paying attention to further environmental effects like land use, each ton of cement thus emits 600–900 kg CO_2_ due to the decomposition of calcium carbonate to calcium oxide, the energy demand, processing and grinding [1,2]. Thus, cement production accounts for 5–8% of the total global anthropogenic carbon dioxide emissions [3,4,5]. While processing techniques in cement mills are permanently optimized, carbon emissions due to calcination cannot be prevented unless the overall clinker content is reduced [6]. Thus, alternative binders and supplementary cementitious materials (SCMs) are the point of interest in ongoing research [7]. Common SCMs are e.g., fly ash (FA), ground blast furnace slags (GBFS), silica fume (SF) and other pozzolanic or latent hydraulic materials, which have been used in many industrialized countries. Meanwhile, these SCMs have no growth potential anymore, as they are mostly already fully being used for cement production, and their global supply is decreasing rather than increasing. State-of-the-art research investigates calcined clay as potentially viable cement replacement materials in emerging economies, due to the huge global clay resources, more simple processing techniques for calcination and lower combustion temperatures [8,9,10]. Clay deposits are enormous potentials on the large scale, particularly in rural areas, where transportation is a major challenge. Still, they do not provide a comprehensive solution regarding increasing construction material demands, due to urbanization and economic development especially in emerging economies.

The world’s population is expected to increase from 7.9 to 9.5–10 billion prospectively by 2050 [11]. Meanwhile, the global cement consumption is expected to increase from an estimated 4.1 Gt/a in 2019 to 6–13.5 Gt/a in 2050 [4,5]. Subsequently, carbon emissions caused by the building sector are about to rise tremendously [12]. According to the Global Cities Institute, by the end of 21st century, 13 out of 20 megacities (metropole regions with more than 10 Mio. inhabitants) will be on the African continent [13]. At the same time, African countries currently record the lowest carbon consumption per capita worldwide, according to Schmidt et al. ([14], data taken from [15,16]). For example, Sub-Saharan Africa (SSA) consumes about 20 times less carbon per capita than the US. The adoption of common building materials, techniques and standards, especially in Africa’s fast growing economies, would increase the global carbon emissions exponentially [17]. Thus, considering the demand of mitigating carbon emissions, further building material developments need to use the advantages of locally available materials, taking the environment, infrastructure and needs of prospective housing into account, to gain sustainable building solutions alongside common norms and Western standards [18].

A proper approach is the use of agricultural by-products as building constituents, which has a positive impact not only on access to and supply of building materials in emerging economies or rural areas, but also on general agricultural waste management [19]. Schmidt et al. present possible climate friendly and low-emission circular value chains from agro-wastes to construction materials as novel value chains for rural development perspectives with an impact on urban development [20]. If properly processed and designed, concrete containing agricultural by-products possess similar or even better properties than traditional concrete [21,22].

Agricultural by-products can be used either as aggregate replacement [23], natural fibers, admixtures or clinker substituents. Ashes from agro-wastes often contain siliceous amounts with pozzolanic or hydraulic properties, i.e., palm fuel oil ashes, bagasse ashes, bamboo ashes or rice husk ashes (RHA). The latter especially have potential as prospective high-performance additions for concrete production. Rice is one of the most important crops in the world: after corn and wheat, it is the third most cultivated grain. In 2016, 756.2 Mio tonnes of unpeeled rice were produced, mostly in Southeast Asia, South America and Africa [24]. After peeling, the husks make out approximately 20% by mass of the whole grain. Thus, rice husks are the largest amount of agro by-products, which is about 151 Mio t globally per year [25]. The husks mostly contain cellulose, lignin and silica. Without processing, they are not usable as animal food or further except for thermal energy use. Meanwhile, the combustion of silica-rich rice husks can lead to ashes with high amounts of amorphous silica and thus pozzolanic activity. If burnt under controlled temperatures, the chemical composition can contain more than 90% of amorphous silica [26]. The proper processing of rice husks to siliceous ash for the use as SCM, which also comprises grinding techniques and microstructural investigation, was investigated by various researchers during the last century [21,27,28]. Especially within the past few years, the performance of RHA as high-performance addition in blended cementitious systems has been investigated more intensively [29,30,31,32].

Despite their potential as high-performance concrete constituent, approximately 115 Mio tonnes of rice husks stay unused agricultural waste annually [33], and at the same time, cement is the most cost-driving factor in Sub-Saharan Africa [34]. In addition to environmental aspects, the design of circular value chains from agro-wastes to building materials could also reduce material costs and, thus, strengthen the wealth of emerging areas, provide prospective sustainable building solutions, creating livelihoods and prevent depositing agricultural wastes.

This paper aims to compare and optimize the combustion and the grinding process of RH under controlled conditions in the laboratory, and easy combustion techniques on the countryside in a self-made furnace, to obtain more knowledge for producing amorphous silica of adequate particle size. Despite the extensive literature regarding the use of RHA as SCM, the direct comparison of one material after different treatments and, thus, possible building material in the field or high quality product after treatment in the lab is missing. Therefore, the study results serve as a basis for further studies on the use of RHA in concrete.

## 2. Previous Studies on the Use of RHA as SCM

Worldwide, there are approximately 22 species of rice with different chemical compositions [33]. With only two species being relevant for human consumption, the chemical composition, i.e., the alkalinity, salinity and acid-sulphur amount, depends on the water and soil supply during cultivation [35]. Thus, not only of the raw material, but also the produced ashes, contain various amounts of trace elements. During combustion, the organic parts of the husks are decomposed. After combustion ideally all organic components are removed, by weight 17–18% of ash remain from the husks [36]. The dominating remaining chemical components are silica (typically more than 85%, up to 98% [37]) and trace elements like potassium, calcium, aluminum, iron and magnesium. Even though the variation of trace elements can affect the final performance of RHA, pozzolanic activity is always given, due to these high amounts of reactive silica.

### 2.1. Combustion, Cooling and Grinding of RHA

Combustion techniques can be either direct or indirect. Direct burning, i.e., when used for energy supply, leads to ashes with varying qualities with regard to the chemical composition and the purity of the silica [36]. The indirect burning of the husks, i.e., combustion in a two-chamber-system, leads to a better control of combustion temperatures and thus more homogenous burning. The quality of these ashes is typically higher. Calcination temperature and heating rate determine the final reactivity, i.e., the pozzolanic index of the RHA [38]. Research on the most appropriate burning temperatures, combustion times and heating and cooling rates often found that appropriate combustion temperatures are between 500 and 800 °C [26,39,40,41,42,43]. Below 500 °C, organic material remains in the ash. Above 800 °C, silica changes its structure from amorphous to crystalline which decreases the pozzolanic reactivity between remaining portlandite (C-H) from the first hydration reaction and silica from the RHA: with a crystalline structure of silica, a pozzolanic phase reaction of strength forming hydrates, i.e., C-S-H formation, is not possible. The heating rate, indeed, affects the porosity of the RHA: Chandrasekhar et al. investigated increasing pore volume with increasing heating rates [38]. Meanwhile, the reactivity increases with increasing porosity, due to higher inner specific surface area and, thus, has a positive effect on the final strength [38], but a negative effect on water adsorption and workability [34,44].

The combustion time depends on the amount of burnt ashes: in most ongoing research, combustion times under controlled conditions are below one hour [28]. On the other hand, Nair et al. reported that combustion temperatures at 1000 °C are possible if the combustion time is less than five minutes [44]. Moreover, the oxidation supply influences the silica form: with little oxidizing conditions, i.e., slow cooling with moderate oxygen supply, the silica appears in amorphous form. A highly oxidizing environment results in crystalline forms of the silica. In summary, the most appropriate combination of combustion temperature and time differs with changing furnace structures and geometries, the volume of burnt rice husks and the processing and control of heating and cooling rates. The production of RHA in controlled laboratories, where indirect burning under controlled temperatures and heating rates is possible in advanced muffle ovens and furnace systems, leads to the purest amorphous structures and, thus, the highest reactivity.

The grinding process determines the fineness of the RHA. With increasing grinding energy and time, the mean particle diameter of RHA decreases. For example, Le describes the decrease in mean diameter from 86.2 µm at 0 min grinding time to 5.7 µm to 540 min grinding time in a ball mill [31]. Due to its structure, sometimes described as a honeycomb structure, the specific surface area is normally 10 to 100 times higher than that of cement, and five to ten times higher than for silica fume [32]. Values from investigation via Brunauer-Emmett-Teller (BET) of properly burnt RHA are in the range of 100–150 m^2^/g [32,45]. With increasing fineness, the inner porosity of RHA decreases. A good overview of effects on chemical and physical composition induced by various parameter variations, such as combustion time, temperature and grinding technique, can be found in Fapohunda et al. [46].

### 2.2. Application of RHA in Practice: From Rural Combustion to High-Performance Concrete

The application possibilities of RHA in practice vary, depending upon existing infrastructure and material supply in different regions in the world, as well as the respective state of the art and research development. Rural combustion techniques offer the possibility of material performance increase, especially in areas where ordinary Portland cement (OPC) is scarce and expensive. Application-oriented furnace-systems besides the lab were invented i.e., by Pitt [47] and Zain et al. [48]: in 1976, Pitt invented a whole furnace system for proper combustion of agricultural wastes in a two-step-approach with a cyclone separator to prevent emerging flue gases. Zain et al. invented a furnace with either one or more steel pipes in the combustion chamber for an effective use of fuels and an exact adjustment of combustion temperatures [48]. Especially the invention by Zain et al. is applicable as an easy combustion method in rural areas. Further similar furnace inventions can be found in [39,42,44].

The subsequent use of RHA burnt under uncontrolled conditions leads to either improved or reduced concrete properties regarding strength and durability, depending on both the characteristics of the pozzolanic activity of RHA, the amount of replacement and the concrete mixture composition itself. The use of uncontrolled burnt RHA often also leads to a decrease in strength and durability properties [49,50,51].

Nevertheless, the production and application of RHA has advanced within the past years. Properly ground RHA, in combination with low w/c ratios and fluidizing admixtures, can lead to a highly reactive blended cementitious system and improved concrete properties [32]. In 2001, Bui developed a material concept for the use of RHA as mineral addition in high performance concrete [45]. In 2011, Nguyen et al. developed this approach further by investigating the use of RHA for ultra-high performance concrete (UHPC) [52]. In 2015, Le investigated especially fresh concrete properties of concrete containing RHA [31]: concerning rheology, Le designed self-compacting concrete (SCC) with RHA. Msinjili et al. investigated rheological properties of RHA blended cement mixtures containing various PCEs [34], whereas Schmidt et al. proposed SCC mixtures making use of the synergistic effect of RHA and limestone filler. While the RHA significantly increased the strength, the workability was drastically reduced. Limestone filler helped to improve the workability, but reduced the strength. A reasonable adjustment allows to significantly reduce the overall Portland cement clinker content without performanceloss. Finally, SCC with RHA was possible by using a combination of lignosulphonate plasticizer and cassava starch as rheology modifying admixtures [53,54].

However, the potentials for applications are lagging behind the research progress in this area: there is no approved and validated common production technique to gain pozzolanic material. Standards for concrete production with RHA do not exist, whereas common SCMs like fly ash or silica fume are mostly regulated all over the world.

The slow progress in implementing RHA in official standards might be caused by inhomogeneities in the material itself, but also the relatively low material amounts annually, compared to the material amounts of other additives. Moreover, big amounts of rice husks grow in countries with relatively low income and little possibilities and inadequate funding for basic infrastructure, further research and development of common building materials. At the same time, cities and communities deal with rapid growth of population and, thus, the need for increased construction and material supply, which leads to relocation, depletion, the emergence of low-income disadvantaged settlements and, consequently, a rapid exploitation of natural resources [55]. Locally available resources need to be investigated, combined with local traditional building techniques and improved for standardization and thus general building applications. RHA as product from a renewable resource can be a proper building material for both, rural production and application and extensive application in concrete production as addition with standardized procedures.

In summary, the production of RHA in research makes it a possible additive in modern concretes like UHPC and SCC. Combustion methods most often can be adopted from existing combustion systems and enhanced for the special needs of RHA. The presented experimental investigations reach to combine both rural combustion and advanced laboratory research to find the gap between the two methods of production and application. The results shall be used as the basis for the development of RHA production and application both for areas with cement supply shortage and low-cost housing and prospective cement reduction possibilities concerning the reduction of carbon emissions in more industrialized economies. These topics can be combined by considering the need for sustainable urban development and ongoing research regarding the circular economy and circular bio-based value chains, especially with the shift from rural to urban application [20].

## 3. Materials and Methods

### 3.1. Concept of Investigation

Rice husks were collected by the authors in Bagamoyo, Tanzania. They were washed with pure water, sundried and not treated further. Combustion possibilities of the rice husks for the application as SCM in concrete were investigated both under rural conditions in Bagamoyo, Tanzania and laboratory conditions in the Center for Building Materials in Munich, Germany. The production of the ashes focused on changing the parameters combustion temperatures and grinding values. The main goal of the investigations was to (1) examine the physical and chemical parameters of produced RHA under controlled and uncontrolled conditions, (2) determine the most appropriate combustion and grinding technique and (3) estimate the application possibilities of controlled laboratory treatment to the field. The physical and chemical parameters of the RHA were investigated using laser granulometry for the investigation of particle size distribution, BET analysis for the estimation of specific surface area, scanning electron microscopy (SEM) for the investigation of the surface structure, inductively coupled plasma optical emission spectrometry (ICP-OES) for the chemical composition and X-ray diffraction (XRD) for the quantitative investigation of amorphous phase content.

In a second step, cement pastes were prepared with varying substitution ratios of cement with RHA or fly ash: in three test series, pastes were prepared with 10%, 20% and 40% RHA by weight of cement (bwoc) cement replacement, respectively. Strength tests were conducted after 28 and 56 days.

### 3.2. Combustion Variation

**Figure 1:**
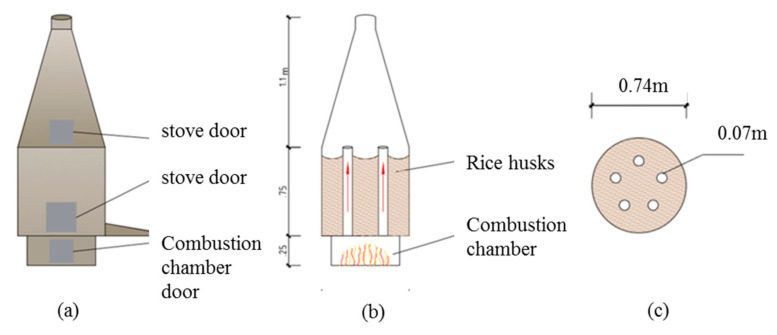
Self-made furnace: (**a**) external view with two stove doors and one combustion chamber door; (**b**) internal view with the combustion chamber and the pipes; (**c**) top view with five pipes.

**Figure 2:**
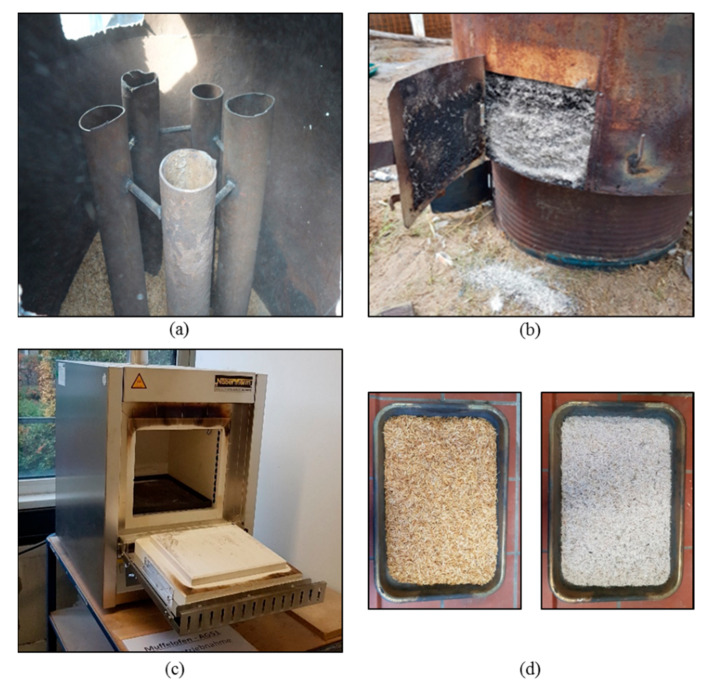
Different combustion methods: (**a**) pipes in a self-made furnace; (**b**) rice husk ash (RHA) after combustion; (**c**) controlled laboratory combustion in a muffle oven and (**d**) RH before (left) and after (right) controlled combustion.

RH were burnt both under uncontrolled conditions with a self-made furnace (Figure 1 and Figure 2a) and for comparison under controlled conditions in a muffle oven in a laboratory (Figure 2c). The sample designation, given by the variation of controlled and uncontrolled combustion conditions, is shown in Table 1.

Combustion under rural conditions took place in a simple self-made furnace, shown in Figure 1: the furnace consisted of a two-chamber system for indirect burning of the husks (Figure 1a). Husks were filled until 0.75 m height in the chamber above the combustion chamber (Figure 1b). Five steel pipes were installed with a diameter of 7.0 cm (Figure 1c). The steel pipes ensured an even heat distribution within the chamber. Fuel could be either charcoal or timber. Charcoal possesses a higher caloric value and, thus, reaches higher temperatures and longer burning times. Tests were conducted with both timber and charcoal: with timber, temperatures around 500 °C were reached. With charcoal, higher constant temperatures between 800 and 1000 °C could be reached. The temperature measurements were conducted with a high temperature thermometer from Professional Instruments, China. Controlled combustion was achieved with a muffle oven in Figure 2c. Temperatures of 450 °C and 650 °C were chosen, following the recommendations of existing studies [30]. For each combustion process, in Figure 2d, only a small amount of husks were burnt, to ensure homogeneous combustion and oxidizing environment. The small sample amount allowed short combustion durations of 30 min, respectively. The husks were only stored in the muffle oven after it reached the combustion temperature. The combustion procedure created homogeneously burnt ash of white color (Figure 2d). After combustion, the RHA was directly cooled down without monitoring the cooling rate.

### 3.3. Grinding Variation

The RHA were ground in a ball mill (Cryomill, Retsch, Germany) with varying grinding times and frequencies. The chosen grinding parameters are given in Table 2. The parameters were adjusted in a stepwise approximation. After grinding, particle size distribution was investigated using laser granulometry with a Mastersizer 2000, Malvern, UK. The specific surface area (SSA) was calculated using the Blaine method. The particle size distribution (PSD) as well as the mean diameter (d_50_) were investigated. Furthermore, the specific surface area (SSA) of the ground RHA was measured by BET nitrogen adsorption method using a Belsorp II Mini (Microtrac BEL, Haan, Germany).

### 3.4. Characterization Methods

Chemical and phase composition was conducted through X-ray diffraction measurements (XRD). The measurements were conducted with a Bruker D8 Advance Diffractometer (Bruker Corporation, Billerica, MA, USA) with Cu Kα radiation and a high-resolution energy-dispersive detector LYNXEYE-XE. The analysis was carried out with automatic divergence slit; 5–70° 2Theta angle range; 0.02° 2Theta step size; 0.2 s measurement time per step; 40 kV voltage and 40 mA current intensity. The resulting peaks were identified based on existing data bases. The Rietveld refinement was used for the quantitative analysis of the results.

XRD can only detect crystalline phases. Accordingly, the amorphous content was investigated by measuring the remaining crystalline amount. The RHA powder for XRD samples was prepared using a ball mill (Cryomill Retsch, Haan, Germany) at 20 HZ and grinding for 20 s. Scanning electron microscopy (SEM) with coupled energy dispersive X-ray spectroscopy (EDS) was conducted on the RHA powder using the FlexSEM 1000 from Hitachi, Japan. ICP-OES analysis using the Avio 500 (PerkinElmer, Germany) finally showed the elementary distribution of RHA.

### 3.5. Compressive Strength Tests: Mixture Composition

For compressive strength tests, cement paste prisms with both pure cement and cement with fly ash of 15 mm × 15 mm × 60 mm were prepared. Cement pastes were mixed containing OPC (CEM I 42.5 R, Heidelberg Cement, Heidelberg, Germany; technical characteristics given in data in brief by Lu et al. [56]) and demineralized water. Fly ash was supplied by Powerment GmbH, Germany. The chemical composition is given in Table 3. The cement was stored at 20 °C without solar irradiation; the added water temperature was 8 °C for a controlled paste temperature at 20 °C. The mixture proportions of all test series for compressive strength tests are given in Table 4. Pure cement paste with a water/cement ratio (w/c ratio) of 0.5 was prepared as reference series (100CEM). Five further test series were conducted. Outgoing from the combustion and grinding analysis, two RHA from the field in Bagamoyo, Tanzania (Uncontrolled U1 and U2) and two RHA after controlled combustion at 450 °C and 650 °C (C450 and C650) were chosen. For an estimation of RHA as SCM in comparison to other pozzolanic SCMs, cement paste containing fly ash (FA) was prepared. For all testing series, the cement amount was stepwise replaced by 10%, 20% and 40% of FA and RHA, respectively. The cement replacement was chosen by the weight of cement for a controlled water/binder ratio (w/b) of 0.5 at all test series. In accordance with different material densities (measured by helium pycnometry, AccuPyc 1330 pycnometer, Micromeritics, Norcross, GA, USA; values are given in Table 3), the fresh paste densities and therefore the hardened paste densities varied.

The pastes were prepared in a bucket with a standard drilling machine and a four-bladed stirrer at a shear of 1700 min^−1^ to ensure a homogeneously mixed paste. For each mixture, a paste volume of 300 mL was prepared. The mixing time was 90 s. The produced cement prisms were stored for one day before striking and subsequently stored for 28 days, respectively, under water at 20 °C room temperature and 65% rel. air humidity. The compressive strength tests were conducted using a testing machine from Walter+Bai, Germany. For each mixture, six cement prisms were tested on compressive strength. The mean values and their standard deviation were calculated from six values for each mixture.

## 4. Results and Discussion

### 4.1. Physical and Chemical Properties of RHA

**Figure 3:**
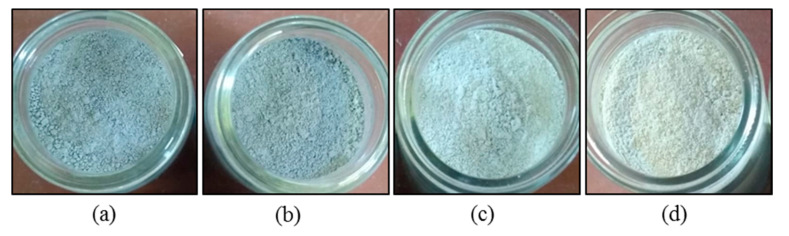
Rice husk ashes after grinding: (**a**) U1, (**b**) U2, (**c**) C450 and (**d**) C650.

The produced RHA are shown in Figure 3a–d. Table 4 shows the material and chemical properties of produced RHA. The density *p* in [g/cm³] was measured by helium pycnometry. The CEM I 42.5 R possesses a mean raw density of 3.11 g/cm³. The density for the RHA varies between 2.25 and 2.32.

**Figure 4:**
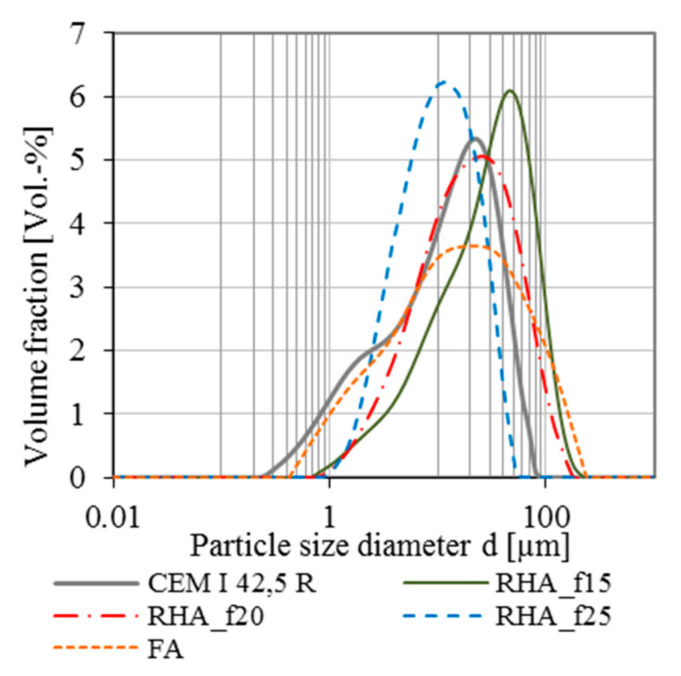
Particle size distribution of CEM I 42.5 R, RHA_f15; RHA_f20, RHA_f25 and fly ash.

The grinding results for different grinding parameters are given in Table 5; the particle size distribution (PSD) for each presented grinding series is given in Figure 4, together with the PSD of cement and fly ash. Whereas RHA_f15 contains higher volume fractions at large particle size diameters, the PSD of RHA_f25 shows a significant shift to the smaller particle size diameters. RHA_f20 possesses similar particle size diameters as the used OPC CEM I 42.5 R. Still, the inner surface of RHA cannot be detected using Blaine method or laser granulometry. Therefore, BET analysis of the ground RHA represents the difference in specific surface area for the used cement and the RHA_f20, considering the inner surface of the material: even if the particle size distribution seems to be similar (cf. Figure 4), the BET for RHA is the tenfold.

**Figure 5:**
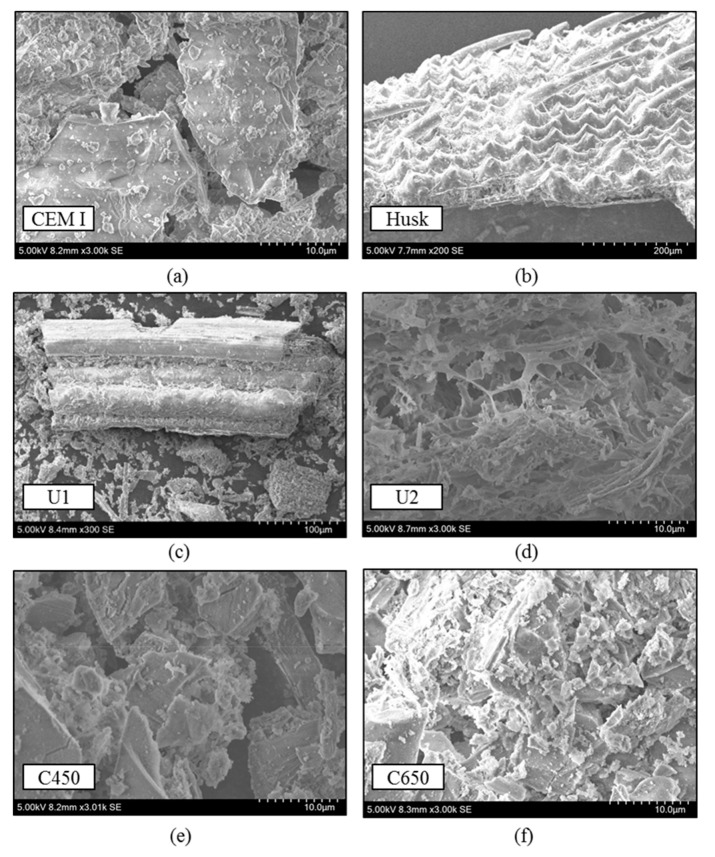
Characteristic scanning electron microscopy (SEM) graphics for: (**a**) CEM I 42.5 R, (**b**) the husks, (**c**) U1, (**d**) U2, (**e**) C450 and (**f**) C650.

SEM graphics show the morphology of cement and the different ground RHA in comparison for magnifications of 3000 SE (Figure 5a–f, except for Figure 5b with a magnification of 200 SE to show the unburnt husk structure and Figure 5c with 300 SE to present the unground RHA structure). Whereas the cement possesses its well-known dense crystalline structure (Figure 5a), RHA is visibly porous. The morphology of the samples U1 and U2 is shown before it was ground. As well as the apparent porosity, especially in Figure 5d, the SEM of U1 in Figure 5c also demonstrates the partly kept husk structure with honey-comb like texture on the husks’ inner surface. Nevertheless, the appearance after proper grinding are given in Figure 5e,f. They present lower porosity but ash patterns and similar outer morphology as cement particles.

**Figure 6:**
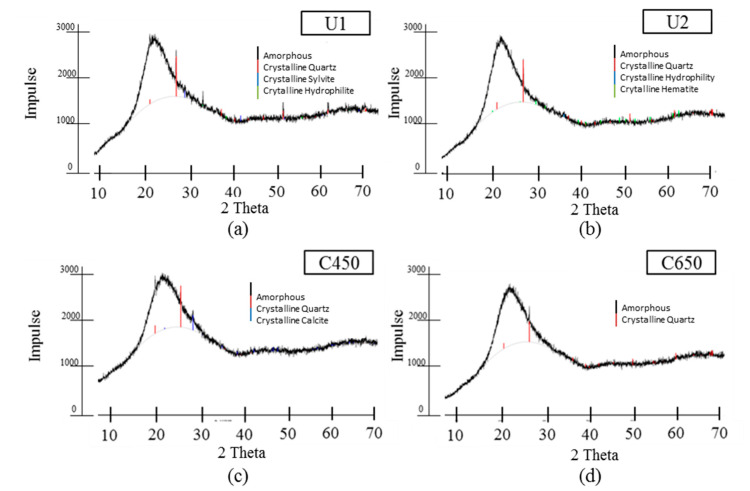
X-ray diffraction (XRD) patterns of RHA samples (**a**) U1, (**b**) U2, (**c**) C450 and (**d**) C650.

SEM-EDS and ICP-OES show the silica phases that are contained in all test samples. The chemical composition from ICP-OES analysis is given in Table 4. The silica content is above 90 M-% for all test samples. C650 contains the highest silica content with about 95% and at the same time very low remaining carbon (0.59%). Even if the C650 contains the highest silica content, the RHA from uncontrolled burning contains only 1% less silica and slightly higher carbon values. For the verification of an amorphous structure of the silica phases, XRD in conducted. The XRD patterns for all RHA samples are given in Figure 6. Except for slight amounts of remaining crystalline quartz and further mineral phases, the whole structure is amorphous. The samples U1 and U2 contain slightly more crystalline phases, with a broader variety of mineral structures.

Comparing the combustion process and the results on the chemical composition, it can be concluded that temperatures around and higher than 450 °C lead to high silica contents if the husks are accumulated, oxidized and burnt homogeneously. Uncontrolled combustion leads to broader chemical distributions within one batch. Controlled combustion, nevertheless, enables a uniform supply of oxygen and, thus, an equal batch with high amorphous silica content.

The presented results show the effect of treatment parameters on the resulting chemical and physical performance. The literature review on the treatment of RHA already described the effect of combustion, grinding and cooling on the performance as SCM. Based on this review of the literature, the treatment parameters for the rice husks were chosen. Generally, the results of the experimental program show a good correspondence with the theoretical background. Particle size distributions similar to cement paste with at the same time high porosity (investigated by BET gas adsorption) could be reached through tuned grinding parameters. With the adjustment of combustion temperature and time, amorphous silica phases were produced. RHA with high amounts of amorphous silica could be obtained if the burning temperature was within a proper range.

### 4.2. Cementitious Paste Results

**Figure 7:**
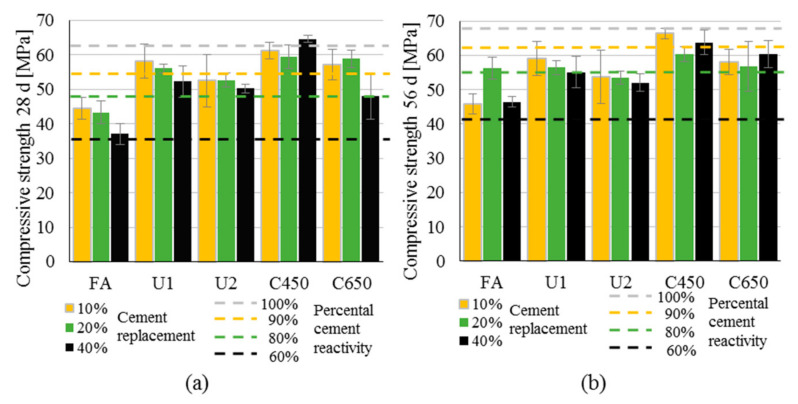
Compressive strength values after (**a**) 28 days and (**b**) 56 days for all test series.

Figure 7 presents the results of compressive strength for all RHA test series after 28 and 56 days compared to pure cement paste results and pastes with fly ash. All values are the average value from six individual tests, and the standard deviation is given as well. The columns in the graphic are the strength values from compressive strength tests. The dotted lines are reference lines regarding the cement activity.

The compressive strength for pure cement paste is given as light grey dashed reference line in Figure 7; the values are 62.5 MPa after 28 days and 69.0 MPa after 56 days. Totals of 90%, 80% and 60% of this measured value are given as reference lines for theoretical cement activity in the test series of RHA with 10%, 20% and 40% cement replacement with RHA, respectively. The FA samples always possess the lowest values. The different test series of RHA show the lowest values for U2 and highest values for C450. Except for the test series C450, which reaches similar strength values as pure cement, all test series show lower results than pure cement paste after 28 and 56 days. Generally, the 28-day strength values for all paste series decreased with increasing cement replacement values with a few exceptions, i.e., 20% replacement RHA in U1 and C650 where the values show slightly increased strength. This is contrary to the results of 56-day strength. Whereas U1 and U2 show decreasing strength values with increasing cement replacement, C450 and C650 show the lowest strength values for 20% replacement.

A comparison of the strength values and the corresponding dotted reference lines of percental cement amount gives an intention of the additional pozzolanic strength gain. Except for the fly ash, nearly all test series with RHA possess higher strength values than a theoretical corresponding mixture with the same amount cement and inert material. Still, there is no overall consistency in cement replacement and strength test results. Generally, 40% replacement lead to a strong decrease in compressive strength, whereas 20% replacement can even improve the performance. At the same time, especially mixtures with 40% RHA show much higher values than their corresponding dashed line of 60% cement—the additional reactivity due to RHA is especially high for C450 and C650. The strength performance of cement with RHA nevertheless is not only dependent on additional strength gain due to pozzolanic activity, but on various additional factors. This material behavior can be explained considering the differing chemical and physical properties. Generally, high silica amounts improve the pozzolanic activity. As could be shown by the chemical analysis, ashes with high purity of amorphous silica and low carbon contents could be reached. C-S-H nevertheless is only formed with remaining portlandite from the first hydration reaction of cement with water. Cement replacement values of 40%, thus, could lead to lower strength, because not all silica from the husks can react with free portlandite. Results of 40% cement replacement, however, could be possible by considering physical factors like increased and optimized packing density compared to pure cement paste, or cement paste with fly ash. A proper amount of fine ground RHA could result in the most appropriate packing density and, thus, the highest reactivity environment.

Finally, it also should be noted that the standard deviations for all test series are quite high compared to standardized mortar or concrete tests. The reason could be either inhomogeneities in the material due to processing, but also the geometry factor of the tested blocks: with only 15 mm × 15 mm × 60 mm, deviations due to boundary conditions might lead to increased scatter.

## 5. Conclusions

The contribution of this paper focuses on the effective treatment and final performance of rice husks under controlled and uncontrolled environments. The investigations present a comparison between uncontrolled and controlled burnt rice husk ashes. The chemical composition of differently burnt husks in dependence of burning conditions and temperature in the field were compared to RHA produced under set conditions in the lab. The grinding process was optimized for rice husk ash compared to cement. Due to its inner porosity, RHA contains the tenfold specific surface area compared to cement under similar particle size distributions.

Comparing the results of RHA production in the field and in the lab, the following concluding remarks can be made:

(1) Rice husk ash production in the field under rural conditions is possible in comparable qualities as controlled laboratory production if the furnace geometry is advanced and production parameters like burning temperature, time, heating and cooling rate and especially heat supply and distribution during the burning are controlled. The grinding value and thus physical characteristics affect the final pozzolanic performance strongly: the extremely high surface area values of RHA lead to more reactive sites for a pozzolanic reaction. Still, its high porosity leads to increased water demand.
(2) The first furnace approach in this study worked out for a proper heat supply for the produced amount of RHA with the system of five steel pipes. Various researchers have investigated and successfully implemented burning systems for agro-wastes, and our very cost-efficient and easy to implement furnace development could show adequate final chemical and physical properties for a pozzolanic ash as well.
(3) Controlled combustion and grinding methods lead to homogeneous RHA with high amorphous silica amounts. With changing material amounts and furnace geometries, these parameters need to be adjusted. In particular, the most effective burning temperature and time needs to be assessed for low-energy production.

Nevertheless, the present investigations contain gaps in efficient production and use of RHA as supplementary cementitious materials. Still, the possibilities for more effective and ecological use both for rural areas and for acceptance as common building material could be shown. Therefore, as a first approach, the following indications can be made:

(a) For efficient energy use, rice husks could be burnt directly. If only low external incinerating energy is needed for a subsequent self-combustion, processing costs and emissions can be reduced. Furthermore, the gained thermal energy from combustion should be stored and supplied e.g., in surrounding households, for continuing the incineration of the RHA furnace or supplied to an outer energy network. Especially for low-income areas, the implementation of a full production line from agro wastes to building materials under energy production will lead to increased wealth.
(b) Rice is globally available and among the most used crops in the world. As renewable resource its husks provide material for the construction sector constantly. Even if the final RHA amounts are not comparable to those of limestone, clays or finally produced clinker, the possibility of each ecologically friendly building material needs to be investigated internationally for common use. This requires international standards and quality production both of RHA and concrete containing RHA. Thus, further investigations need to be made regarding strength, and especially durability performance.

Finally, the investigations regarding the proper treatment of rice husks and the subsequent use of RHA as SCM could show good results, especially regarding the performance of RHA produced in the field. Ongoing research focuses on both microstructural and application improvements. Performance tests according to existing standards together with material development might lead to both better knowledge about various rural application methods, as well as high-performance material and admission and thus broad use as prospective concrete addition. Various leading institutions, like RILEM, fib, etc., recently described the urgent need of sustainable material solutions for the growing demand of concrete [57]. RHA could be one of them.

## Figures and Tables

**Table 1:** Combustion temperatures.

Sample Name	Combustion Temperature	Combustion Time (h)	Cooling Time (h)
U1 ^1^	500	03:00	24:00
U2 ^2^	>800	03:00	24:00
C450	450	00:30	00:30
C650	650	00:30	00:30

^1^ The rice husks RHA_UC1 were burnt with timber. ^2^ The rice husks RHA_UC2 were burnt with charcoal.

**Table 2:** Grinding values.

Sample Name	Frequency (Hz)	Time of Grinding (sec)
RHA_f15	15	10
RHA_f20	20	20
RHA_f25	25	40

**Table 3:** Physical and chemical material properties.

Sample Name	Specific Gravity *ρ* (g/cm^3^)	SiO_2_ (%)	Al_2_O_3_ (%)	Fe_2_O_3_ (%)	CaO (%)	MgO (%)	Na_2_O (%)	K_2_O (%)	TC (%)	XRD: Amorphous Phase Content (%)
**U1**	2.25	93.78	0.48	0.72	2.07	0.52	0.38	1.89	0.88	>99.9
**U2**	2.32	93.90	0.20	0.43	0.84	0.37	0.38	1.69	0.56	>99.0
**C450**	2.25	93.15	0.36	0.26	0.60	0.37	0.43	1.81	0.66	>98.8
**C650**	2.29	94.97	0.97	0.58	0.83	0.57	0.58	1.66	0.59	>99.5
**Fly ash**	2.30	55.54	20.76	11.26	3.88	1.38	0.84	2.09	-	-

**Table 4:** Cement paste mixtures.

Sample Names	w/b Ratio	Cement (kg/m^3^)	SCM [-]	SCM Amount (kg/m^3^)	Water (kg/m^3^)	Fresh Paste Density (kg/m³)
100CEM	0.5	1217.2	-	-	608.6	1830
90CEM_10FA	0.5	1090.5	10%	109.1	599.8	1690
80CEM_20FA	0.5	987.7	20%	197.5	592.6	1580
60CEM_40FA	0.5	831.0	40%	332.4	581.7	1410
90CEM_10C450	0.5	1091.7	10%	109.2	600.4	1690
80CEM_20C450	0.5	989.7	20%	197.9	593.8	1580
60CEM_40C450	0.5	833.8	40%	333.5	583.7	1420
90CEM_10C650	0.5	1092.6	10%	109.3	601.0	1690
80CEM_20C650	0.5	991.2	20%	198.2	594.7	1590
60CEM_40C650	0.5	836.0	40%	334.4	585.2	1420
90CEM_10U1	0.5	1092.6	10%	109.3	601.0	1690
80CEM_20U1	0.5	991.2	20%	198.2	594.7	1590
60CEM_40U1	0.5	836.0	40%	334.4	585.2	1420
90CEM_10U2	0.5	1091.7	10%	109.2	600.4	1690
80CEM_20U2	0.5	989.7	20%	197.9	593.8	1580
60CEM_40U2	0.5	833.8	40%	333.5	583.7	1420

**Table 5:** Grinding values.

Sample Name	Blaine SSA (cm²/g)	PSD (µm)	d_50_ (µm)	BET SSA (m²/g)
CEM I 42.5 R	4300	0.3–100	15.0	1.24
RHA_f15	1920	1.0–50.0	32.0	-
RHA_f20	2690	0.8–260.0	20.0	128
RHA_f25	3880	0.9–200.0	9.5	-

Blaine SSA: Blaine specific surface area; PSD: particle size distribution; d_50_: mean diameter of the particle size distribution; BET SSA: specific surface area measured by BET adsorption method.
